# Glucose tolerance and markers of myocardial injury after an acute coronary syndrome: predictive role of the 1-h plus 2-h plasma glucose at the oral glucose tolerance test

**DOI:** 10.1186/s12933-022-01590-w

**Published:** 2022-08-08

**Authors:** Viola Zywicki, Paola Capozza, Paolo Caravelli, Stefano Del Prato, Raffaele De Caterina

**Affiliations:** 1 Cardiology, University of Pisa, and Cardiovascular Division - Pisa University Hospital, Via Paradisa, 2, 56124 Pisa, Italy; 2 Diabetology Divisions, Pisa University Hospital, University of Pisa, Pisa, Italy; 3 Fondazione VillaSerena Per La Ricerca, Città Sant’Angelo, Pescara Italy

**Keywords:** Diabetes, Impaired glucose tolerance, Oral glucose tolerance test, Cardiovascular risk, Acute coronary syndromes

## Abstract

**Objective:**

Impaired glucose tolerance (IGT) has been related to adverse cardiovascular outcomes. We investigated the added value of 1-h plasma glucose (PG) at the oral glucose tolerance test (OGTT) in predicting admission and peak cardiac high-sensitivity troponin T (hs-TnT) and NT-proBNP values in IGT patients admitted for an acute coronary syndrome (ACS).

**Research design and methods:**

Among 192 consecutive ACS patients, 109 had Hb1Ac and fasting plasma glucose negative for newly diagnosed diabetes. Upon OGTT performed > 96 h after admission, 88, conventionally diagnosed as IGT, were divided into: “full glucose tolerance” (1-h PG-OGTT < 155 mg/dL and 2-h PG-OGTT < 140 mg/dL, N = 12);”early IGT” (1 h-PG-OGTT ≥ 155 mg/dL and 2-h PG-OGTT < 140 mg/dL, N = 33);**”**late IGT” (1-h PG-OGTT < 155 mg/dL and 2-h PG-OGTT ≥ 140 mg/dL, N = 8); and “full IGT” (1-h PG-OGTT ≥ 155 mg/dL and 2-h PG-OGTT ≥ 140 mg/dL, N = 35). The 4 groups were compared for cardiac markers.

**Results:**

The first three groups had similar cardiac marker values, but only full IGT patients had significantly higher admission hs-TnT compared with the 3 other groups [median (interquartile range): 911 (245-2976) vs 292 (46-1131), P < 0.001]. Full IGT patients also had higher hs-TnT peak compared with fully glucose tolerant and early IGT patients. Only full IGT patients had longer hospitalization and higher NT-proBNP vs fully glucose tolerant patients (P = 0.005).

**Conclusions:**

Among non-diabetic ACS patients, only those with both 1-h PG ≥ 155 mg/dL and 2-h PG ≥ 140 mg/dL had more severe myocardial injury and longer hospitalization. One-h PG-OGTT importantly contributes to assessing post-ACS cardiac risk.

**Supplementary Information:**

The online version contains supplementary material available at 10.1186/s12933-022-01590-w.

## Introduction

Type 2 diabetes is a metabolic disorder characterized by persistent hyperglycemia, due to impaired insulin secretion and insulin resistance, leading to micro- and macrovascular complications. “Prediabetes” identifies a condition characterized by altered fasting plasma glucose (PG) (100–125 mg/dL), glycated hemoglobin (HbA1c) 5.7–6.4%, or impaired glucose tolerance (IGT, with a 2-h oral glucose tolerance test (OGTT) featuring blood glucose 140–199 mg/dL). It has been shown that the OGTT is a more sensitive screening tool than HbA1c for the detection of previously unrecognized glycemic disorders in patients with acute stroke with an at least a 25% relative difference in detection rate [1]. On the other hand, in two thirds of the patients admitted with acute myocardial infarction (AMI) with no known history of diabetes, an HbA1c in the prediabetes range, but not the OGTT, added predictive value on the long-term outcome [2]. It has also been reported that type 2 diabetes screening by means of an algorithm combining FPG and 1-h PG limits the demand of a 2-h OGTT in 79% of CAD patients without known diabetes [3]. Thus, the additive role of the OGTT in characterizing cardiovascular risk is still currently under debate. In addition, 1-h OGTT PG levels > 155 mg/dL not primarily and universally reported in the results of the OGTT, have been suggested to confer a higher risk of developing type 2 diabetes and cardiovascular disease [4–7]. In the GEN-FIEV study, individuals with normal glucose tolerance (i.e., 2-h PG-OGTT < 140 mg/dL) but with 1-h PG-OGTT > 155 mg/dL were more prone to develop diabetes and had a worse cardiovascular risk profile compared with individuals with 1-h PG-OGTT ≤ 155 mg/dL [8]. Moreover, in the CATAMERI study, 1-h PG-OGTT ≥ 155 mg/dL has been associated with a pro-atherogenic profile [6, 9], and patients with prediabetes identified by Hb1Ac threshold and 1-h PG-OGTT ≥ 155 mg/dL had significantly higher prevalence of cerebrovascular disease and coronary artery disease compared with those with 1-h PG < 155 mg/dL [10]. Thus, the additive role of OGTT-derived parameters in characterizing cardiovascular risk is still worth investigation.

The prevalence of undiagnosed dysglycemia in people admitted to hospitals for an acute coronary syndrome (ACS) is high [11–13], and is associated with poorer in-hospital [14] and long-term outcomes [13], with significantly higher cardiovascular morbidity and mortality compared with normoglycemic subjects. There is therefore the need for a screening of dysglycemia after an ACS to detect unknown diabetes or identify prediabetes, due to its relevance for cardiovascular risk. Such a screening is supported by data [15] and currently recommended by guidelines [16], but not widely adopted in common clinical practice in the acute or subacute setting, both for practicality reasons (reported data from Italian coronary care units, unpublished) and because also increasingly discouraged [17, 18]. Even fasting PG and HbA1c are not commonly considered in investigating borderline patients in acute conditions because of the concern that they might improperly reflect the stress condition induced by the acute coronary event.

Because of these uncertainties, we here aimed at thoroughly investigating the role of the OGTT in stratifying cardiovascular risk early after an ACS, particularly focusing on the relevance of the 1-h OGTT-PG ≥ 155 mg/dL.

## Research design and methods

### Patient population

From May 2020 to May 2021 all consecutive patients admitted to the coronary care unit of Pisa University Hospital for an ACS and fulfilling the inclusion and exclusion criteria of the study were prospectively enrolled. ACS included, as per the European Society of Cardiology definition, unstable angina, non-ST-elevation myocardial infarction (NSTEMI) and ST-elevation myocardial infarction (STEMI).

We excluded patients aged < 18 years, those with a former diagnosis of either diabetes or pre-diabetes [19], with cardiogenic shock entailing an immediate risk of death, or with cognitive dysfunction precluding the possibility of an informed consent to participate in the study. In order to avoid confounding factors possibly limiting data interpretation, we also excluded patients with renal failure requiring chronic hemodialysis or patients with class III renal dysfunction (estimated glomerular filtration rate (eGFR) < 30 mL/min calculated from plasma creatinine with the Chronic Kidney Disease Epidemiology Collaboration (CKD-EPI) equation.

During the hospital admission and from clinical records we obtained information about demographics, cardiovascular risk factor, lifestyle habits and medications. After the hospital discharge, at 3 and 12 months a standard outpatient evaluation, including additional examinations and evaluations as demanded by the clinical status, was performed, as routinely done for all patients at our center. This study is to be considered preliminary to a larger one assessing the relevance of OGTT-derived parameters on hard cardiovascular outcomes. Because of its preliminary nature and of the unknown prevalence of OGTT parameters derangements in this specific population and settings, we did establish a precise sample size estimate. In addition, this study being an analysis of data prospectively acquired through the OGTT routinely performed in our Cardiology Division for clinical purposes and not entailing any additional intervention on patients, the Ethics Committee approval was not strictly required and we did not not seek it.

### Baseline characterization: venous blood sampling, transthoracic echocardiography and coronary artery disease characterization

Upon admission to the coronary care unit, a baseline evaluation of complete blood cell count and differential, thyroid hormones, lipid profile, liver and renal function (serum eGFR) calculated from plasma creatinine based on the CKD-EPI equation, fasting plasma glucose, HbA1c, serial high-sensitivity troponin T (hs-TnT), N-terminal pro B-type natriuretic peptide (NT-proBNP), BNP and C-Reactive Protein (CRP) were obtained in all participants from a venous blood sampling. A 2-Dimensional echocardiographic examination with both pulsed-wave and continuous-wave color-Doppler was performed at least twice, within 24 h since admission and before discharge, to obtain the left ventricular ejection fraction as an evaluation of myocardial impairment. Echocardiographic examinations were performed and revised by trained cardiologists in the echocardiography laboratory of our Cardiology Unit, with training and performances certified by the Italian Society of Echocardiography and Cardiovascular Imaging (Società Italiana di Ecocardiografia e Cardiovascular Imaging, SIECVI). Specifically, the inter-observer variability in the assessment of ejection fraction has been internally estimated to be < 8.5%. The ejection fraction assessment nearest patient discharge was here considered for the analyses. All subjects underwent coronary angiography and, if suitable, a percutaneous coronary intervention, within 24 h from admission.

Individuals with fasting plasma glucose and HbA1c not diagnostic for diabetes underwent a fully standardized 75 g OGTT (Glucosio Sclavo Diagnostic^®^, Sclavo Diagnostic International) after an overnight fasting. Venous blood samples were drawn at baseline, 1 h and 2 h after oral glucose administration (with an additional sample drawn at 1.5 h in the first 88 cases). All OGTTs were performed after at least 96 h after admission.

## .

### Statistical analysis

For all quantitative parameters examined, normal distribution was assessed with the Kolmogorov–Smirnov test. As all continuous variables had a non-normal distribution, they were expressed as medians and interquartile intervals. Differences between groups were evaluated through the Kruskal–Wallis Test, and multiple comparisons corrected with the Bonferroni’s adjustment. Categorical variables were compared by the Chi-square test. Two-tailed P values < 0.05 were considered statistically significant. All statistical analyses were performed using the IBM SPSS statistical package (version 22, 2013).

## Results

### Patient population

From May 2020 to May 2021, a total of 192 patients fulfilling the enrollment criteria were admitted to the coronary care unit of Pisa University Hospital, with an ACS as admission diagnosis (51% STEMI, 25% NSTEMI, 11% unstable angina). Their glycemic status was first evaluated with fasting plasma glucose and HbA1c. Among these patients, 118 had no former diagnosis of either diabetes or pre-diabetes, and 109 had Hb1Ac and fasting PG results either negative or inconclusive for a new diagnosis of diabetes. In all these patients, an OGTT was performed > 96 h after admission and usually just before discharge. On the basis of the OGTT results, 21 patients (19.3%) were thus considered as having newly diagnosed diabetes, and this group was not further considered in the analyses.

The remaining 88 patients, qualifying for the current investigation, were divided into 4 Groups on the on the base of the OGTT results:

1. Group A, fully normoglycemic patients: 12 patients (13.6%), with 1-h PG-OGTT < 155 mg/dL and 2-h PG-OGTT < 140 mg/dL;
2. Group B, “early IGT”: 33 patients (37.5%) with 1-h PG-OGTT ≥ 155 mg/dL and 2-h PG-OGTT < 140 mg;
3. Group C, “late IGT”: 8 patients (9.1%) with IGT and 1-h PG-OGTT < 155 mg/dL;
4. Group D, “full IGT”: 35 patients (39.8%) with IGT and 1-h PG-OGTT ≥ 155 mg/dL.

Patient disposition is reported in the Additional file 1: Figure S1, and main characteristics of patients included in the study are summarized in the Additional file 1: Table S1. Patients’ overall median age (IQR) of the four groups was 63.5 (54.0–72.0) years, and age was comparable across the groups of interest. Patients were more often male (77.5%) than female, and the most common diagnosis was STEMI (59.1%), followed by NSTEMI (22.7%). Few patients had a diagnosis of MI with normal coronary arteries (MINOCA) or the Tako-Tsubo syndrome (Additional file 1: Table S1).

Cardiovascular risk factors were common, particularly hypercholesterolemia (69.3%) and current or former smoking (60.2%) There were no statistically significant differences among groups regarding these risk factors, as there was no difference in the prevalence of arterial hypertension and family history of coronary artery disease across the 4 groups (Additional file 1: Table S1).

Interestingly, there were no statistically significant differences in Hb1Ac values. FPG values were significantly higher in Groups B, C and D vs Group A, but not significantly different in Groups B, C and D. In all, since abnormal FPG values were an exclusion criterion for the enrollment, baseline FBG values were below the diabetic threshold (Additional file 1: Table S1).

### Glycemic profiles and in-hospital outcome

**Fig. 1:**
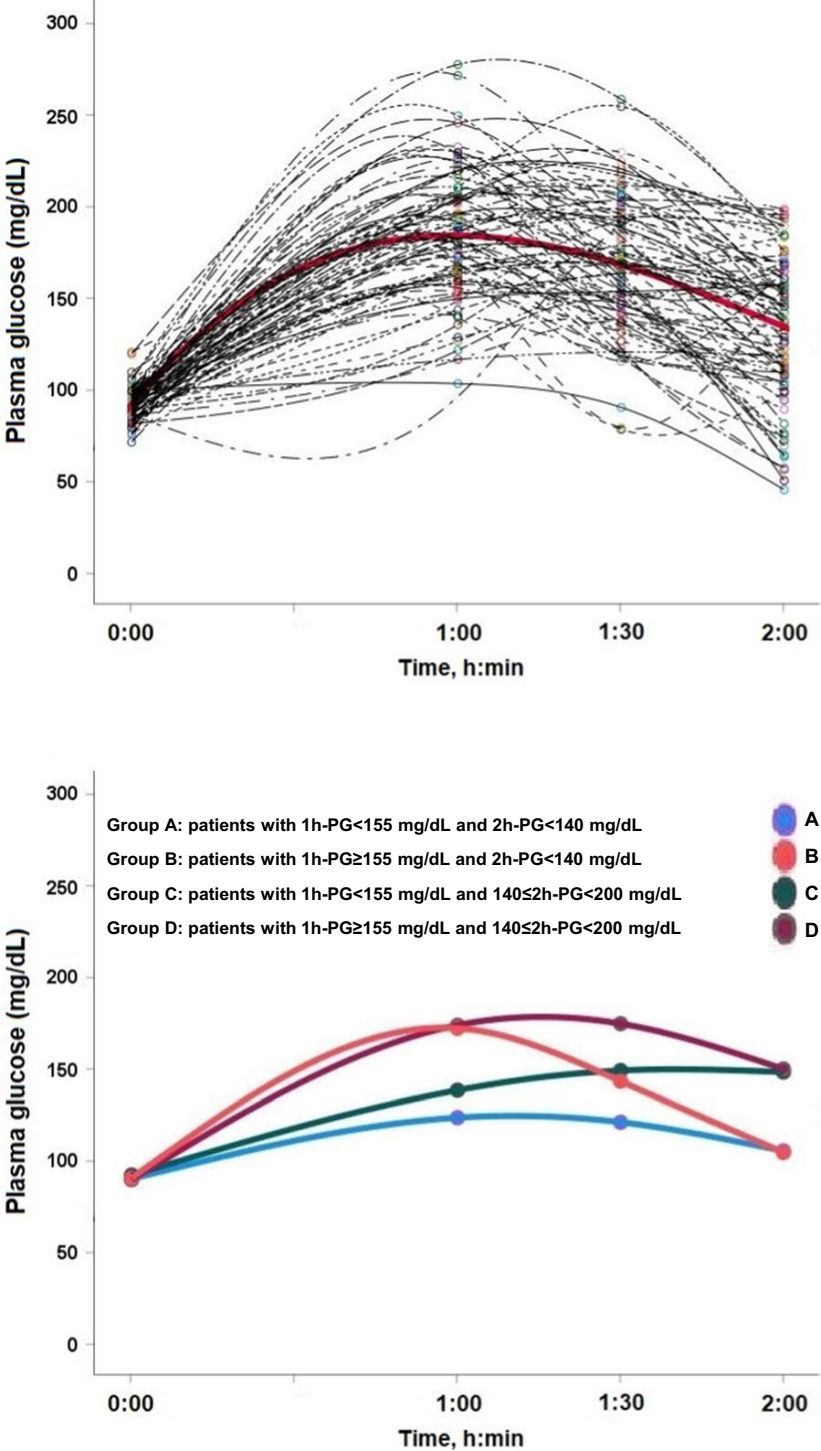
Upper panel: Time course of plasma glucose levels during the oral glucose tolerance tests (OGTT) in the 88 patients tested at baseline (0 h) and at 1, 1.5 and 2 h. Lower panel: Time course of median plasma glucose (PG) levels during the oral glucose tolerance tests (OGTT) of the four groups tested at baseline (0 h) and at 1, 1.5 and 2 h

Figure 1 shows the interpolated time course of plasma glucose levels following the OGTT in all patients included in the study. Most subjects had a 1-h PG-OGTT > 155 mg/dL, with much greater variability after that time point (upper panel). Median values of plasma glucose in the four groups are shown in the lower panel of Fig. 1.

**Fig. 2:**
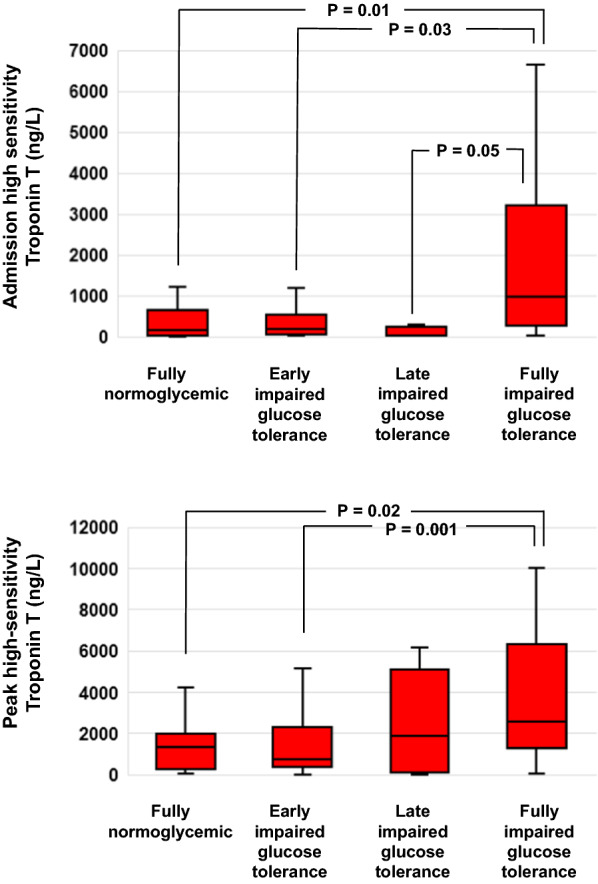
Box plot of admission (upper panel) and peak (lower panel) high-sensitivity troponin T (hs-TnT) distributions on the base of previously defined OGTT groups. Box plots display the 25^th^, the 50^th^ (median) and the 75^th^ percentiles in the box, and whiskers display the minimum and the maximum of the data set. Horizontal brackets join groups for which statistically significant differences (P < 0.05) were found at multiple comparisons

Both serum levels hs-TnT at admission (P = 0.0001) and peak hs-TnT (P = 0.003) were statistically different across the 4 groups (Table 1). This is also illustrated in Fig. 2, showing how Group D –i.e., full IGT patients—had higher admission hs-TnT compared with all the 3 other groups. As to peak hs-TnT, conversely, no difference was apparent between Groups D—full IGT patients—and C—i.e., late IGT.

**Table 1:** In-hospital outcome measures: cardiac biomarkers and cardiac function parameters in the four patient groups

	All Patients n = 88	Group A n = 12	Group B n = 33	Group C n = 8	Group D n = 35	P-value
Hospitalization duration (daya)	6.0 (5.0–7.0)	5.0 (4.2–5.7)	6.0 (5.0–7.0)	6.5 (5.0–7.7)	7.0 (6.0–8.0)	0.02*
LVEF admission (%)	51 (44–58)	55 (53–59)	51 (44–58)	50 (40–58)	51 (42–55)	0.08
NT-proBNP (ng/L)	689 (277–1348)	370 (124–770)	671 (311–1301)	220 (122–700)	1123 (551–2686)	0.001**
BNP (pg/mL)	99 (38–197)	45 (16–172)	61 (33–133)	123 (38–172)	148 (78–378)	0.12
Admission hs-Troponin T (ng/L)	292 (46–1131)	42 (21–375)	166 (57–472)	29 (10–235)	911 (245–2976)	0.001***
Discharge hs-Troponin T (ng/L)	251 (97–658)	284 (63–661)	155 (35–259)	18 (49 -1120)	399 (202–850)	0.056
Peak hs-Troponin T (ng/L)	1331 (355–3743)	522 (175–1495)	654 (232–1694)	1853 (87–5063)	2113 (1327–7483)	0.003****

Group A: patients with normal glucose tolerance and 1 h-PG < 155 mg/dL; Group B: patient with normal glucose tolerance and 1 h-PG ≥ 155 mg/dL; Group C: patients with IGT characterized by 1 h-PG < 155 mg/dL; Group D: patients with IGT including 1 h-PG ≥ 155 mg/dL

Two-tailed P-values < 0.05 were considered as statistically significant. Continuos variables were expressed with median and interquartiles intervals

*hs-Troponin T* high-sensitivity Troponin T, *LVEF* left ventricular ejection fraction, *OGTT-PG* oral glucose tolerance test plasma glucose, *PG* plasma glucose

^*^ = Statistically significant multiple comparisons: A vs D: P = 0.005

^**^ = Statistically significant multiple comparison: A vs D: P = 0.008, C vs D: P = 0.015

^***^ = Statistically significant multiple comparison: A vs D: P = 0.013, B vs D: P = 0.023, C vs D P = 0.005

^****^ = Statistically significant multiple comparisons: A vs D: P = 0.024, D vs B, P = 0

**Fig. 3:**
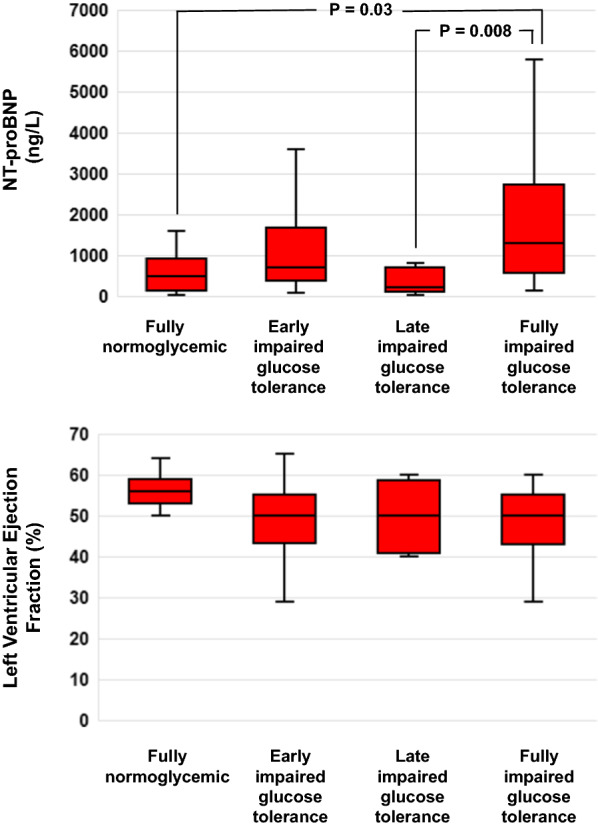
Box plot of admission N-terminal prohormone of the brain natriuretic peptide (NT-proBNP, upper panel) and admission % left ventricular ejection fraction (LVEF) distributions on the base of previously defined OGTT groups. Box plots display the 25th, the 50th (median) and the 75^th^ percentiles in the box, and whiskers display the minimum and the maximum of the data set. Horizontal brackets join groups for which statistically significant differences (P < 0.05) were found at multiple comparisons

Group D—full IGT patients—also had higher NT-proBNP values when compared with Group A [1123 ng/L (551–2686) vs 370 ng/L (124–770) (P = 0.008)] and Group C [1123 ng/L (551–2686) vs 220 ng/L (122–700) (P = 0.015)] (Fig. 3). Group D featured the nominally lowest left ventricular ejection fraction values, although not different from the other groups to a statistically significant extent (Fig. 3). Patients with full IGT also had the numerically highest BNP (Table 1) and aspartate amino transferase levels (Additional file 1: Table S1).

As reported in Table 1, Group D had longer hospitalizations compared with patients in Group A [7.0 days (6.0–8.0) vs 5.0 days (4.2–5.7) (P = 0.005)]. At a median follow up of 9 months, 4 subjects had major adverse cardiovascular events (MACE): one stroke, one death for cardiogenic shock, two readmissions for ACS requiring percutaneous coronary revascularization. Two of these events occurred in Group B, 1 in Group C and 2 in Group D. No cardiovascular event occurred in fully normoglycemic subjects.

Since group C was small (n = 8) and, similarly to group D, had a high 2-h PG-OGTT, by pooling the two groups in a sensitivity analysis we confirmed that subjects with high 2-h PG-OGTT were characterized by the same risk profile as Group D (data not shown).

## Discussion

We here report that, in patients admitted to a coronary care unit because of an ACS, the OGTT including both 1-h and 2-h PG values identifies patients with the most severe in-hospital risk profile, adverse remodeling and longer hospitalization; and that the addition of the 1-h PG-OGTT to the usually assessed 2-h PG-OGTT is of help in such identification.

Despite the previously reported high percentage of undiagnosed dysglycemia among ACS patients [20] and the relevant prognostic role of 2-h-PG at the OGTT [21] in the coronary care setting, the use of glucose challenge has been increasingly neglected or actually discouraged [17, 18]. We have here re-evaluated the importance of OGTT in providing prognostic information with respect to the severity of myocardial injury and remodeling, as well as to mid-term outcomes. Our results broadly confirm that IGT at the time of hospital discharge associates with worse “outcomes”, in our case investigated with markers of myocardial injury. In our case, however, it is IGT also including elevated 1-h-PG at the time of hospital discharge to be associated with high levels of NT-proBNP and troponin, as well as a numerically lower LVEF and longer hospitalization.

After a myocardial infarction, the in-hospital diagnosis of IGT through the performance of the OGTT had already demonstrated to provide important long-term prognostic information: IGT is indeed associated with the increased occurrence of MACE in the follow-up [13, 22]. In the acute phase, irrespectively of the presence of diabetes, a hyperglycemic status is associated with a higher risk of in-hospital death, cardiogenic shock and congestive heart failure, and correlates with a more extensive myocardial damage [23], This association likely reflects a higher degree of inflammation and the concomitant release of counterregulatory hormones [23, 24], resulting in a higher degree of stress hyperglycemia, a condition that has been associated with poorer outcomes in a number of acute conditions, including myocardial infarction [23, 25, 26], stroke [27], trauma [28, 29] and, more recently, COVID-19 [30]. In this sense, our report confirms previous findings on the prognostic role of the OGTT in patients with an acute myocardial infarction.

We have here also explored, however, to what extent 1-h OGTT PG may also have a prognostic value, both as an isolated defect (i.e., 11-h OGTT PG > 155 mg/dL and 2-h OGTT PG < 140 mg/dL), as well as in combination with IGT (i.e., 1-h OGTT PG > 155 mg/dL and 2-h OGTT PG > 140 mg/dL). Previous work had indeed suggested the 1-h OGTT PG may be a simpler and more effective criterion for identifying people at risk of developing type 2 diabetes, cardiovascular disease and death [6, 8–10, 31, 32]. To the best of our knowledge, however, no studies has so far investigated the prognostic potential of 1-h OGTT PG at the time of hospital admission because of an ACS. Our results suggest that, although 1-h OGTT PG may identify people with a more severe cardiac injury and impaired remodeling, it is full IGT that best associates with a worse risk profile. Thus, patients with isolated 1-h IGT had a risk profile that was not different from those with normal glucose tolerance and better than in patients with the combination of the two glucose tolerance defects. This latter finding suggests that a more comprehensive glucose tolerance disturbance, assessed by alterations of both the 1-h and the 2-h PG at the OGTT, best identifies patients with admission and discharge conditions more severe than those in patients with isolated altered 2-h PG. Of note, indeed, patients with full IGT featured higher peak TnT and NT-proBNP values than late IGT patients, i.e., those still conventionally and now broadly classified as with “IGT”. The same full IGT individuals were the ones with a worse left ventricular ejection fraction and longer hospitalization. Because the latter group was small in size, and we could therefore not exclude lack of power, a sensitivity analysis performed by pooling group C and group D confirmed the overall findings.

We recorded new cardiovascular events over 9 months after discharge, but this analysis remains largely exploratory given the relatively small size of each of the glucose tolerance group and the very limited number of events (N = 4). Yet, it is intriguing that 1-h-altered PG patients apparently had no more events compared with those with normal glucose tolerance. Our report, therefore, while broadly confirming older data on the usefulness of the routine safe performance of the OGTT in patients otherwise classified as non-diabetic to refine the prognostic stratification of ACS [15, 20], points to the importance of accruing data on the shape of the OGTT curve.

We acknowledge several limitations in this report, which cannot be considered conclusive. First, the limited sample size: this has to be regarded as the first analysis of this kind, with no a priori effect size postulated and no sample size estimated. In fact, the data here accrued will serve as the basis for a larger, more definitive study, now ongoing. A second limitation is in the non-homogenous distribution of patients among groups might have prevented to document differences among groups beyond those here reported. As shown in Table 1, Group A, B and C patients had absolute lower levels of NT-proBNP, peak and admission hs-TnT values compared with group D patients. Other parameters, however, failed to achieve statistically significant differences at multiple comparisons. In particular we did not observe any statistically significant difference in left ventricular ejection fraction values despite Group D featured the numerically lowest values. For the majority of patients (79.5%), this was the first in-hospital access for an ACS: in these patients, we observed a relevant undertreatment of cardiovascular risk factors: particularly, despite a high prevalence of dyslipidemia, only 15.7% of patients reported daily statin treatment; in particular, in Group D no patient reported any statin treatment at admission. This was likely a chance finding, but can theoretically influence the outcome..Third, we considered laboratory markers, instrumental examinations and hospitalization duration instead of clinical events to evaluate in-hospital outcomes. The choice of surrogate markers of disease severity was dictated by the small sample size and the short duration of the follow-up. Further studies including larger populations will be required to determine to what extent the 1-h OGTT PG may contribute independent of and beyond the prognostic value of 2-h PG. Recruitment of a larger patient cohort with a longer-term follow-up is currently ongoing. At the moment we hypothesize that in-hospital outcomes will be later reflected in adverse outcomes in terms of major adverse cardiovascular events at a longer follow-up, but this will have to be verified. Fifth, we cannot definitely rule-out a carry-over effect of the ACS on the outcomes of the OGTT. We feel, however, that a later evaluation would carry little practical implication for the difficulty of implementation after discharge. Groups were also here too small to detect differences between STEMI and non-ST elevation ACS.

Finally, our findings are at variance from those obtained in a primary prevention setting, where 1-h PG at the OGTT was shown to correlate with the later development of diabetes [4, 5] and adverse cardiovascular outcomes irrespective of the 2-h PG [6]. In our cohort, we failed to demonstrate any difference among Group A and Group B patients, letting us to conclude that, in our setting, the sole 1-h PG with the current cut-off of 155 mg/dL should not be considered as a sufficient substitute for the standard 2-h PG at the OGTT, but rather as potentially yielding complementary information.

In summary: in an ACS setting, the routine use of the OGTT including the 1-h PG evaluation before discharge helps identifying, among newly diagnosed IGT, a subset of patients experiencing adverse in-hospital outcomes. Further research should confirm these data in a larger cohort and assess their translation into harder outcomes.

## Supplementary Information

**Additional file 1: Table S1.** Demographics and baseline characteristics in the four patient groups**. Figure S1**. Patient enrollment and disposition

## Data Availability

Available upon reasonable request.
