# Supplementary material for: Glucose tolerance and markers of myocardial injury after an acute coronary syndrome: predictive role of the 1-h plus 2-h plasma glucose at the oral glucose tolerance test
Source: Cardiovasc Diabetol. 2022 Aug 8;21:152. doi: 10.1186/s12933-022-01590-w (PMC9358640; doi:10.1186/s12933-022-01590-w)
Supplement: Supplementary file 1 — Additional file 1: Table S1. Demographics and baseline characteristics in the four patient groups. Figure S1. Patient enrollment and disposition [file 12933_2022_1590_MOESM1_ESM.docx]

**Glucose Tolerance and markers of myocardial injury after an acute coronary syndrome – Predictive role of the 1-h plus 2-h plasma glucose at the oral glucose tolerance test**

Short title: 1-h plus 2-h plasma glucose at the OGTT and myocardial injury markers

Viola Zywicki, MD^1^, Paola Francesca Capozza, MD^1^, Paolo Caravelli, MD, PhD^1^,

Stefano Del Prato, MD^2^, and Raffaele De Caterina, MD, PhD^1, 3^

^1^Cardiology and ^2^Diabetology Divisions, Pisa University Hospital, University of Pisa and ^3^Fondazione VillaSerena per la Ricerca, Città Sant’Angelo, Pescara, Italy

**Online Supplement**

**Address for correspondence:**

Prof. Raffaele De Caterina, MD, PhD

Chair of Cardiology, University of Pisa, and Cardiovascular Division - Pisa University Hospital, Via Paradisa, 2 - 56124 Pisa, Italy

Tel.: +39-050-996-751

E-mail: [raffaele.decaterina@unipi.it](mailto:raffaele.decaterina@unipi.it)

**
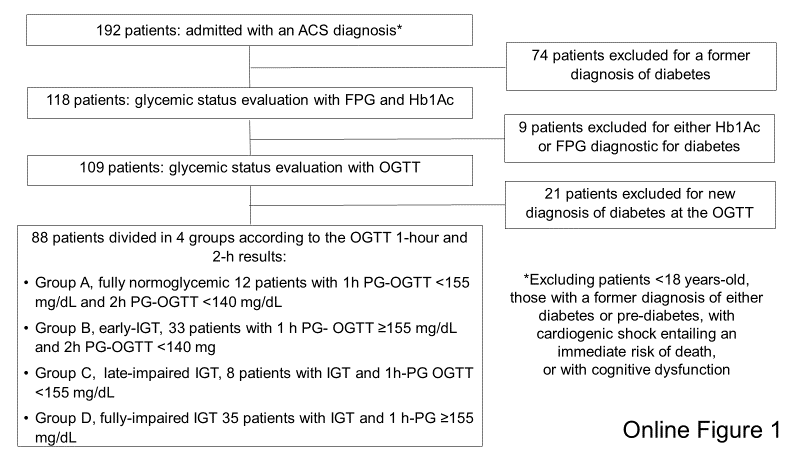
**

**Online Figure 1: Patient enrollment and disposition.** Abbreviations: ACS, acute coronary syndrome, FPG, fasting plasma glucose; IGT, impaired glucose tolerance; OGTT, oral glucose tolerance test.
